# Mortality among People Living with HIV and AIDS in China: Implications for Enhancing Linkage

**DOI:** 10.1038/srep28005

**Published:** 2016-06-21

**Authors:** Meng Li, Weiming Tang, Kai Bu, Tanmay Mahapatra, Xiayan Zhang, Yibing Feng, Fangfang Chen, Wei Guo, Liyan Wang, Zhengwei Ding, Qianqian Qin, Shiliang Liu, Joseph D. Tucker, Lu Wang, Ning Wang

**Affiliations:** 1 National Center for AIDS/STD Control and Prevention, Chinese Center for Disease Control and Prevention, Beijing, China; 2 University of North Carolina Project-China, Guangzhou, China; 3 University of California, Los Angeles, CA, USA

## Abstract

To assess the patterns and predictors of AIDS-related mortality and identify its correlates among adult people living with HIV/AIDS (PLWHA) in China, a retrospective record-based cohort study was conducted among 18 years or older PLWHA, who had at least one follow up reported to the national database between January-1989 and June-2012. Cumulative Incidence Function was used to calculate AIDS-related mortality rate. Gray’s test was used to determine the variation in cumulative incidence across strata. The Fine and Gray model was used to measure the burden of cumulative incidence of AIDS-related mortality and strength of its association with potential correlates. Among 375,629 patients, 107,634 died during study period, of which 54,759 (50.87%) deaths were AIDS-related. Cumulative mortality rates of AIDS-related death at one, two, five, 10 and 15 years post-diagnosis were 5.7%, 8.2%, 14.3%, 22.9% and 30.9%, respectively. Among PLWHA, male gender, ethnic minority and having AIDS were associated with significantly higher mortality. Further, homosexual transmission, being on ART and increasing CD4-testing frequency were associated with lower mortality. To reduce mortality among PLWHA, efficient interventions targeting males, ethnic minority, heterosexually infected and AIDS patients should be combined with immunologic monitoring, enhancement of coverage of HIV-testing and ART.

Despite remarkable progress in promotion of access to HIV prevention, treatment and supportive care, HIV epidemic in China continued to expand. At the end of the year 2011, an estimated 780,000 people were living with HIV/AIDS (PLWHA) in this country, about 48,000 persons became newly infected with HIV during this year and the number of reported HIV cases were increasing over years^1^. Without an effective cure or vaccine, mortality attributable to HIV also remained a major public health concern in China. For example, the number of HIV/AIDS-related deaths increased from 5544 in 2007 to 21,234 in 2011 in this country^1^. Especially, in 2008, HIV/AIDS became the leading infectious cause of death, killing nearly 7000 people during the first nine months of that year^2^,^3^.

Despite the well-established positive role of preventive approach in containment of the HIV epidemic, in China, much of the national response so far had only focused on ensuring greater access of PLWHA to antiretroviral treatment (ART). To strengthen the treatment and follow-up involving this population, the “National Free Antiretroviral Treatment Hand book” was issued in 2011with revised criteria for initiation of treatment, prioritizing drug resistance testing and management of switching drug regimens. These efforts cumulatively resulted in an increase in the estimated number of people currently receiving ART from 65481 in 2009 to 126448 in 2011 and those on ART were reported to have better likelihood of survival^1^,^4^.

Though PLWHA could expect a longer life expectancy by virtue of timely treatment and effective control of opportunistic infections^5^,^6^,^7^,^8^,^9^, given the limited access to ART, rapid emergence of drug resistance, increasing complications, poor retention rates and expensive second line of treatment, the effectiveness of early initiation and increased coverage of ART alone in minimizing AIDS-related deaths remained debatable. Evidence suggesting upsurge of the HIV epidemic and associated deaths resulted in stronger political commitment to raise public awareness regarding HIV and to promote HIV prevention in China. With the aim of 25% and 30% reduction, respectively, in HIV incidence and mortality by 2015, at the end of 2010, Government of China implemented “Five Expands, Six strengthens” policy involving information-education-communication (IEC) activities, surveillance and testing, prevention-of-mother-to-child-transmission (PMTCT), comprehensive interventions and coverage of ART^1^. Despite sincere efforts, only 42% of the eligible residents of this country were estimated to have received ART at the end of 2011^1^.

Incomplete and inaccurate population level data on HIV mortality were additional points of concern in China. Burden and predictors of mortality among PLWHA remained understudied here owing to several logistic factors^10^. Based on a study in recent past the overall mortality rate among PLWHA in China diminished from 39.3/100 person-years in 2002 to 14.2/100 person-years in 2009^11^. However, information on annual mortality rate among PLWHA, since the time of diagnosis and reporting were unavailable. Additionally, there is a paucity of data pertaining to the factors related to HIV related death in Chinese context as most of the previously published literatures only documented the effect of ART on mortality among HIV infected people across the country^11^,^12^. Comprehensive understanding of the predictors of mortality in this gradually enlarging population was not only essential for accurate evaluation of HIV scenario in China but appeared also to be crucial for strategic planning of targeted responses, based on local epidemic situations^13^.

The dynamics of mortality among HIV infected persons is complex and a function of many socio-demographic factors, routes of infection, disease status at the time of diagnosis and the quality of the received treatment^14^. Hence the dearth of information regarding the determinants of mortality among PLWHA in China called for a detailed retrospective national level investigation to assess the impact of HIV on adult mortality and to identify correlates of total and AIDS-related mortality among adult PLWHA in this country.

Our study aimed to evaluate the mortality rate among PLWHA since they were identified/reported and to evaluate the potential correlates of AIDS related and unrelated deaths in this population in China, by analyzing the data from a concurrent cohort study (The National HIV Epidemiology Cohort) which was monitoring mortality among PLWHA in China.

## Method

The data used in this current article were obtained from the HIV/AIDS case reporting system (CRS) under the National Center for AIDS/STD Control and Prevention of the Chinese Center for Disease Control and Prevention (China CDC) between 1989 and 2013. The methods were carried out in accordance with the approved guidelines.

### Recruitment

Detailed information regarding the relevant databases is described elsewhere^15^. In brief, this retrospective cohort study was based on Chinese HIV/AIDS case report system and treatment database. Any information collected from these two platforms was included in the current study base, while the two systems were linked by a unique personal ID. No additional identification information was collected from the participants. All newly identified HIV cases were reported to the web-based systems either by local hospitals or clinics. Information on demographic characteristics [age, gender, occupation, ethnicity, address, registered place of residency (Hukou) etc.], HIV related risk-behaviors, treatment history, routes of transmission (heterosexual/homosexual/IDU/transfusion of blood or other blood cells) and disease status (HIV/AIDS based on WHO criteria) at the time of diagnosis were also collected from all the registered PLWHA.

PLWHA were considered eligible to be recruited for this concurrent cohort study if they were aged 18 years or older and had at least one follow up record since their initial reporting to the national database between January 1, 1989 and June 30, 2012. Frequency of CD4 testing for each participant was also calculated in every six months, and frequency of CD4 testing was defined as the cumulative number of CD4 testing at each year divided by two (every six months).

### Follow up

After identification and reporting, all HIV cases were followed up by the local CDCs. The intervals of two follow up varied between three or six months, depending on the disease status. If already been progressed to AIDS, the patients were followed in every three months, otherwise, they were followed six monthly. Accordingly, during the follow up period, blood samples for CD4 count and viral load testing were collected in every 3 or 6 months from each patient. The patients were appropriately treated as per the criteria. Treatment was indicated for confirmed sero-positive WHO Stage III or IV clinical HIV cases and those who had any of the following: symptomatic disease, extra-pulmonary tuberculosis (TB), laboratory criteria of CD4 count below 350 cells/μl or in the absence of CD4 count results: total lymphocyte count below 1200 cells/μl)^16^. The treatment criteria did change over time. Before 2007, only those PLWHA who had progressed to AIDS and had CD4 count <200 cells/μl were considered eligible for treatment. This cut-off for CD4 count changed to ≤350 cells/μl in 2007 and to ≤500 cells/μl in 2014^17^. If the patient received treatment, s/he was also reported to the Treatment Reporting System (TRS). In case of any death during the follow up period, the time and reason of death were recorded.

HIV/AIDS related mortality rate was estimated using the number of deaths among the cases within each follow-up period as the numerator and the cohort’s total person-years at risk within each follow-up period as the denominator. For those who died, half of the follow-up duration (between 2 follow-ups) was used as their contribution to the total person-time at risk.

During follow up period, if one patient was died, the reason of death will be put into the follow up system. Per ICD 10, if the patients were died of AIDS, AIDS related opportunistic infections, AIDS-related tumors or AIDS-related syndrome, their death were coded as AIDS related death, otherwise, their death were coded as Non-AIDS related death.

### Data analysis

The National HIV Epidemiology Cohort was retrospectively analyzed to calculate the mortality rate and to identify factors associated with death among PLWHA in China. During the pulling of the data from the case report and treatment databases, all personal identifiers were removed before the data analysis.

SAS version 9.4^18^ was used for all statistical analyses. Descriptive analyses were conducted to determine the distribution of demographic factors, possible transmission routes [homosexual, heterosexual, injecting drug users (IDU), professional donation of blood or blood products (blood cell), transfusion of blood or blood products, sexual and IDU both routes together, others or unidentified] and outcomes (survived, dead or lost to follow up).

As depending upon the disease status (AIDS patients or Non-AIDS patients (HIV carriers) follow up and CD4 testing frequency varied over time (for AIDS patients, CD4 testing was conducted every 3 months, for HIV carriers, CD4 testing is conducted twice/year), cumulative number of previous CD4 tests were calculated and disease status was assessed at every 6 mouths. Both of these parameters were thus regarded as time-varying risk factors.

Bias due to competing risks could arise in this study if an event of failure in treatment would have resulted from one of the several causes and one of them precluded the others^14^,^15^,^16^. Thus, two groups of competing risks models were built, by using AIDS-related deaths and non-AIDS-related death as event, respectively. Cumulative Incidence Function (CIF) was used to calculate AIDS-related mortality rate of the HIV/AIDS patients during the follow up period. The Gray’s test^17^ method was also used to determine the variation in cumulative incidence across the strata of treatment status, gender and possible transmission routes. The model proposed by Fine and Gray^18^ which was based on the hazard of the sub-distribution was used to measure the strengths of association between cumulative incidence of AIDS-related and non-AIDS-related mortality and its potential correlates (such as baseline demographic factors, possible transmission routes, disease status and whether received ART or not) among the recruited PLWHA. The results were expressed as a hazard ratio (HR) and corresponding 95% confidence interval (95% CI) both for bivariate [to determine individual associations, expressed as crude (HR)] and multivariable [to determine adjusted associations while controlling for all other covariates, expressed as adjusted (aHR),] analyses, while demographic characteristics including age, gender, education, marital status, nationality and occupation were adjusted for in the multivariate models. Observations for which P values were less than 0.05 were considered to be statistically significant. Multivariable models were checked for any multi-collenearity but no such issue was found.

### Ethics statement

The study protocol, contents and procedures were reviewed and approved by the Institutional Review Board of the National Center for AIDS Prevention and Control, China CDC. Written informed consent was obtained from participants at the recruitment stage.

## Results

### Study participants

**Figure 1:**
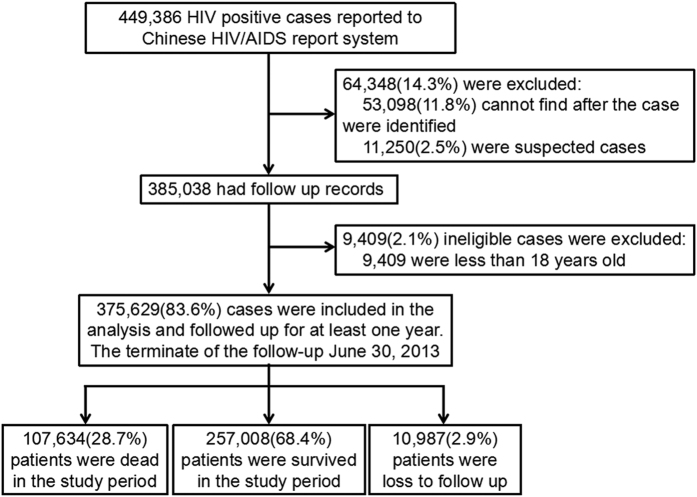
Flow chart of the recruitment among HIV-infected individuals in China 1989–2013 (N = 375,629).

Between January 1, 1989 and June 30, 2012, a total of 449,386 HIV positive cases were reported to the Chinese HIV/AIDS report system. Among these cases, 64,348 (14.32%) were excluded either due to lost contact after being reported to the systems (53,098 cases, 11.82%, having no follow-up records), or because of the lack of confirmation of the HIV sero-positivity by Western Blot testing (11,250 cases, 2.50%). Further, another 9,409 (2.09%) patients were excluded as they were aged less than 18 years (Fig. 1). Overall, 375,629 HIV/AIDS patients were included in the current analyses.

About 70.0% of the eligible patients were male, 64.6% were aged less than 40 years during diagnosis while approximately 73.0% were ever married (18.6% were divorced or widowed). About three quarters (73.7%) of the patients belonged to Han nationality, and about half of the patients reported that they were farmers. In addition, about 40.0% of the eligible patients were either illiterate or attended elementary school only (Table 1).

When identified, about half of the participants already progressed to AIDS. For about half of the subjects, the possible transmission route was heterosexual, and 48% of the patients were already on ART (Table 1).

### Outcomes

At the end of the study follow up period (June 30, 2013), among the recruited PLWHA, 10,987 (2.9%) were lost to follow up, 107,634 (28.7%) died, while the other 257,008 individuals were surviving. Among these 107,634 deaths during the study period, 54,759 (50.9%) were categorized as HIV/AIDS-related death, while the other 52,875 died from reasons (e.g. suicide, drug overuse, unknown or others) other than HIV (Fig. 1).

**Figure 2:**
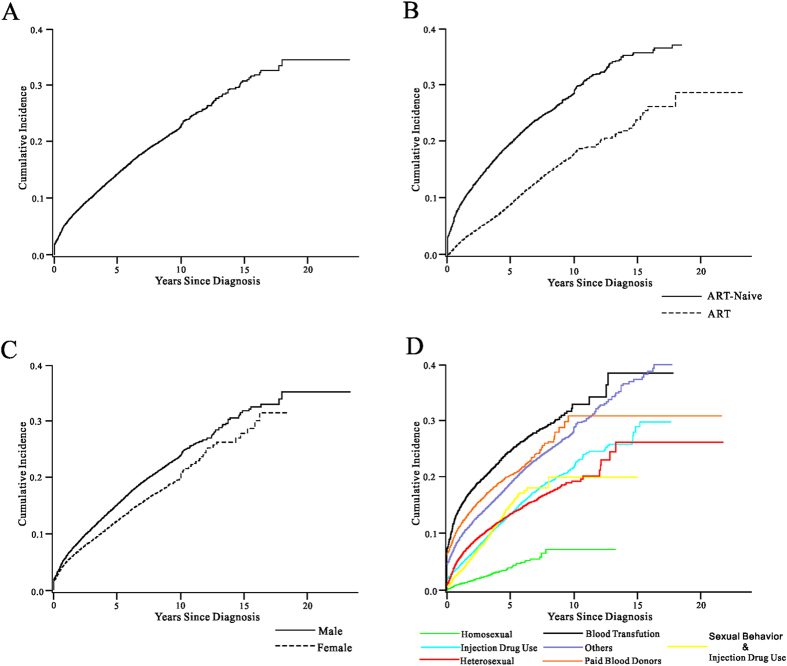
AIDS-related mortality rates among HIV-infected individuals in China 1989–2013 (N = 375,629). (**A**) the overall AIDS-related mortality rate; (**B**) the AIDS-related mortality rates for patients who received and did not receive ART; (**C**) the AIDS-related mortality rates for male and female; (**D**) the AIDS-related mortality rates of patients infected through different transmission routes.

The mortality rates of AIDS-related death at one, two, five, 10 and 15 years after the cases were identified were 5.7%, 8.2%, 14.3%, 22.9% and 30.9%, respectively (Fig. 2A, Table 2). Males and patients who were ART-naive during recruitment had significantly higher mortality rates (P < 0.001) (Table 2).

**Figure 3:**
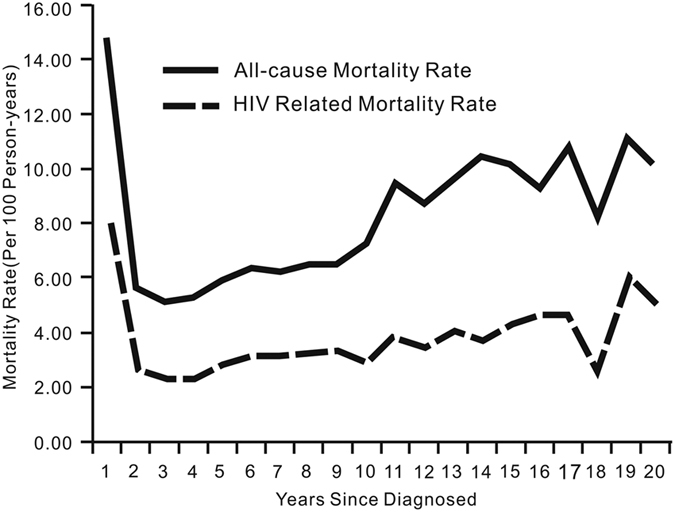
Mortality Rate of Chinese HIV/AIDS patients, 1989–2013 (N = 375,629)

Mortality rate of the participants at each follow up year was presented in Fig. 3. Since few cases were followed up for more than 20 years, the cumulative morality rate was calculated for the first twenty years only. Mortality rates for all deaths and AIDS-related deaths were highest in the first year of follow up, dropped to lowest during the third year and then gradually increased thereafter with in-between drops. During the 20^th^ post-identification year, the mortality rates for all deaths and AIDS-related deaths reached 10.17/100 PYs and 5.08/100 PYs respectively.

**Figure 4:**
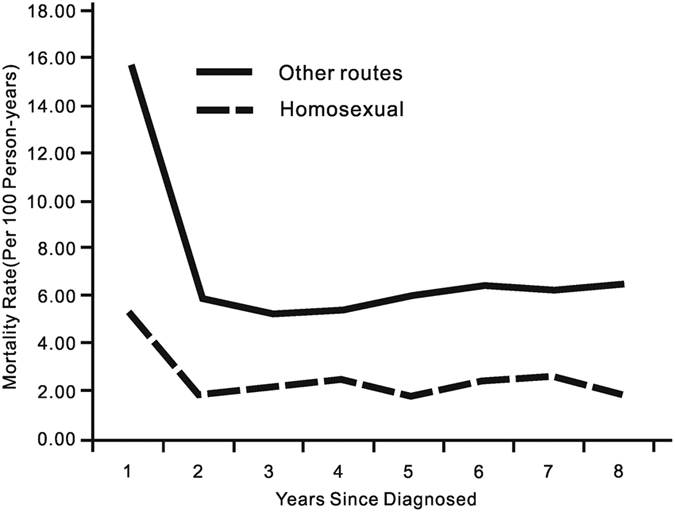
Mortality Rate of Chinese HIV/AIDS patients who were infected through homosexual contact or through other routes, 1989–2013 (N = 375,629).

Current study did also show that the mortality rates among subjects infected through homosexual route were much lower than the overall mortality rate in the follow up period (Fig. 4, adjusted for age). The mortality rate among homosexuals was calculated for the first eight years because few individuals infected through homosexual route were followed for more than eight years.

### Results of competing risks model

In the competing risks model with the outcome of AIDS-related mortality, it was found that patients who belonged to ethnic minority groups had a significantly higher mortality rate (compared to Han), with aHR of 1.21 (1.17–1.25) for Uygur, Zhuang, Yi or Dai and 1.20 (95% CI = 1.14–1.25) for others (other 51 ethnicity minority groups in China). Patients who had progressed to AIDS had significantly higher mortality rate than those who had not (aHR 7.42, 95% CI = 7.21–7.64). Compared to those who got infected through heterosexual route, homosexuals had a significantly lower AIDS-related mortality rate (aHR 0.56 95% CI = 0.52–0.62). Being on ART was also associated with 65% reduction in the hazard of AIDS-related mortality among HIV patients (aHR 0.35, 95% CI = 0.34–0.36). In addition, it was also observed that those whose CD4 testing frequency increased one time per 6 months was associated with 81% reduction in the hazard of AIDS-related mortality (aHR 0.19, 95% CI = 0.17–0.20) (Table 3).

Regarding non-AIDS-related deaths, AIDS (aHR 1.17, 1.14–1.20) were significantly associated with higher mortality rates. Uygur/Zhuang/Yi/Dai, aHR 0.86, 0.84–0.89) and other ethnic minority groups other than Uygur/Zhuang/Yi/Dai (aHR 0.88, 0.84–0.92) were significantly associated with lower mortality rates. Being on ART was also associated with 71% reduction in the hazard of non-AIDS-related mortality among HIV patients (aHR 0.29, 95% CI = 0.27–0.30). In addition, it was also observed that those whose CD4 testing frequency increased one time per 6 months was associated with 80% reduction in the hazard of AIDS-related mortality (aHR 0.20, 95% CI = 0.17–0.23) (Table 4).

## Discussion

In China, the majority of studies on HIV mortality conducted till date only explored the influence of ART and related operational indicators^11^,^12^,^19^. In this record based retrospective cohort study, the highest morality rates for AIDS-related death and all-cause death were found in the first year of follow up after HIV diagnosis. This phenomenon could be explained by the fact that about half of the participating cases had already progressed to AIDS before being identified, as the results of the current study also supported that progression to AIDS as one of the strongest risk factors for AIDS-related death, with an aHR of 7.42.

Mortality rates during the first follow up year and results of competing risks model suggested that HIV patients who got infected through homosexual contact had a significantly lower rate of AIDS-related mortality compared to heterosexually infected patients. This finding corroborated with the observation from a previous study conducted in Denmark^20^, but was inconsistent with several others studies where the progression of HIV/AIDS was found to be faster among homosexuals than subjects infected through other routes^21^. Potential explanations included the fact that owing to the recent upsurge of the HIV epidemic and increased coverage of HIV testing since 2008 among men who have sex with men (MSM), most of the recruited HIV positive MSM were probably in the early stages of the disease progression^22^ as 94% (31,095 out of 32958) of them were identified as a case of HIV between January 1, 2008 and June 30, 2012. It was also observed that, among PLWHA, MSM (compared to those infected through other routes) were significantly younger, well educated, more frequently tested for CD4 count and had higher treatment rate as well as better adherence for the provided care (Supplementary Table 1)^11^,^19^.

This study also indicated that the overall survival rate dropped substantially at 20 years post-diagnosis. As few people were followed for a span of more than 20 years this observed reduction in the survival could be considered as a result of sparse data problem, although negative biological influence of long-standing HIV infection should also be borne in mind.

It was also found that among PLWHA in China, minority populations had significantly higher AIDS-related mortality rates than HIV patients with Han ethnicity. A parallel scenario was also observed among African Americans compared to Caucasians in the USA^23^. Poor education, lack of knowledge regarding HIV/AIDS, lower social economic status and poor access to health care could be the main reasons for this, as these patients from minority ethnicities were mostly living in rural areas.

In this study, about half of the deaths were not related to AIDS. This probably indicated that in order to reduce the overall mortality among HIV patients, additional attention should be paid to the causes of death other than those traditionally been considered to be AIDS-related^24^.

It appeared that more frequent CD4 testing was associated with prolonged survival. This finding was concurrent with the results of a systematic review which reported that clinical and immunologic combined monitoring (include CD4 testing) was better than clinical monitoring alone in terms of a combined mortality and morbidity endpoint^25^. This finding suggested that CD4 testing/monitoring should be performed consistently, which was one of the recommendations of the 2013 WHO ART guidelines^26^.

There were some limitations in this study. It was not possible to include CD4 count and viral load in the analysis because before 2003, HIV patients of China were rarely tested for CD4 count and viral load, and the routine use of these tests started only since 2010. Future sub-analysis should deal with this shortcoming, by limiting the analysis among the patients who got tested at baseline for CD4 count and viral load. As some data elements were based on self-report (e.g. the routes of transmission), current study might have suffered from some miss-classifications. Outcome misclassification, particularly misclassification between AIDS-related and unrelated death, could be another problem, as the outcomes were reported by different hospitals leading to non-standardized ascertainment of the cause of death. Most of the MSM patients were identified in recent years, hence the short follow-up period was a barrier for the complete analyses regarding the progression of HIV to AIDS in this population. Moreover, being a record-based study, this current investigation captured limited information of the patients; hence the scope to adjust for potential confounders was also limited. Beside these, identified number of patients at each year was used as a surrogate for the number of PLWHA who got tested for HIV; this number could have been influenced by both the epidemic situation and the coverage of testing. Last but not the least, both ART and CD4 count being time- dependent variables in nature, due to the missing data problem and the complexity of treatment regimes, we could not properly treat them as time-dependent, which might have resulted in the potential for some bias in the currently reported results.

Even with these limitations, the results of this study revealed that a large proportion of PLWHA in China was suffering from the issues pertaining to very late diagnosis, and mortality rate in this population was very high. Among these patients, ethnic minority, male gender, AIDS and not receiving ART were associated with higher AIDS-related mortality. While targeted interventions to expand HIV testing services and coverage of ART should continue with emphasis, urgent attention to address the risk factors for non- AIDS-related mortality among HIV patients seemed to be the need of the hour in this country. In the era of universal treatment, more implementation research focusing on the promotion of HIV testing and case finding, reduction of the barriers of treatment, and enhancement of the treatment coverage and retention in care rate (particular for minority and rural people) were needed urgently.

## Additional Information

**How to cite this article**: Li, M. *et al.* Mortality among People Living with HIV and AIDS in China: Implications for Enhancing Linkage. *Sci. Rep.* **6**, 28005; doi: 10.1038/srep28005 (2016).

## Supplementary Material

Supplementary Information

## Figures and Tables

**Table 1:** Demographic characteristics, possible transmission routes and treatment status of the HIV/AIDS patients in China, 1.1.1989–06.30.2013 (N = 375,629).

Variables		Number of patients	Percentage (%)
Gender	Male	261865	69.71
	Female	113764	30.29
Age	18-	112863	30.05
	30-	129606	34.50
	40-	69453	18.49
	50-	33685	8.97
	60-	30022	7.99
Marital Status	Married	203353	54.14
	Never married	92033	24.50
	Widowed or divorced	69985	18.63
	Unknown	10258	2.73
Nationality	Han	276910	73.72
	Uygur/Zhuang/Yi/Dai	69649	18.54
	Others	23773	6.33
	Unknown	5297	1.41
Occupation	Farmer	184742	49.18
	Housekeeping, housework and unemployed	64471	17.16
	Worker	21306	5.67
	Business	15817	4.21
	Migrant workers	11013	2.93
	Government staff	9636	2.57
	Retired	8291	2.21
	Others or unknown	60353	16.07
Education	Illiteracy	33533	8.93
	Elementary	118204	31.47
	Junior high school	149217	39.72
	Senior high school	42099	11.21
	College or above	25389	6.76
	Unknown	7187	1.91
Disease status when identified	HIV	190135	50.62
	AIDS	185494	49.38
Possible transmission route	Heterosexual	189766	50.52
	IDU	83972	22.36
	Blood sell	39354	10.48
	Homosexual	32958	8.77
	Blood transfusion	11244	2.99
	Sexual + IDU	4662	1.24
	Others or unknown	13673	3.64
Treatment	No	196119	52.21
	Yes	179510	47.79

**Table 2:** Estimated Cumulative Incidence Functions of the mortality of AIDS-related Death for Chinese HIV/AIDS patients, 1.1.1989–06.30.2013 (N = 375,629).

		Years Since Diagnosis					
		1	2	5	10	15	*P*
Variables		mortality rate (95%*CI*, %)	mortality rate (95%*CI*, %)	mortality rate (95%*CI*, %)	mortality rate (95%*CI*, %)	mortality rate (95%*CI*, %)	
Overall		5.73 (5.65–5.80)	8.16 (8.07–8.26)	14.26 (14.12–14.40)	22.93 (22.40–23.48)	30.88 (28.93–32.84)	
Treatment status	Not treated	9.09 (8.95–9.23)	12.37 (12.21–12.54)	20.10 (19.85–20.35)	29.60 (28.66–30.54)	36.10 (34.18–38.02)	<0.001
	Treated	2.48 (2.41–2.55)	4.20 (4.11–4.30)	9.25 (9.08–9.42)	18.25 (17.60–18.90)	24.74 (21.95–27.63)	
Gender	Male	6.02 (5.93–6.12)	8.63 (8.52–8.75)	15.19 (15.01–15.37)	24.19 (23.55–24.85)	32.17 (29.91–34.46)	<0.001
	Female	4.95 (4.82–5.09)	7.04 (6.88–7.20)	12.31 (12.01–12.55)	20.89 (19.84–21.96)	28.79 (24.98–32.70)	

**Table 3:** Fine and Gray model (hazard of the sub-distribution model) for AIDS-related Death.

Variables		AIDS-related Death	
		Crude HR (95%CI)	Adjusted HR (95%CI)
Nationality	Han	Ref	
	Uygur/Zhuang/Yi/Dai	1.21 (1.18–1.25)	1.21 (1.17–1.25)
	Others	1.19 (1.14–1.24)	1.20 (1.14–1.25)
Disease status^©^	HIV	Ref	
	AIDS	2.53 (2.47–2.60)	7.42 (7.21–7.64)
Possible Transmission route	Heterosexual	Ref	
	IDU	1.08 (1.05–1.12)	0.93 (0.90–0.96)
	Blood sell	0.87 (0.84–0.90)	0.87 (0.83–0.90)
	Homosexual	0.33 (0.31–0.36)	0.56 (0.52–0.62)
	Blood transfusion	1.38 (1.31–1.45)	1.29 (1.22–1.37)
	Sexual+IDU	1.15 (1.04–1.26)	0.91 (0.82–1.00)
	Others or unknown	1.54 (1.46–1.62)	1.17 (1.11–1.24)
Treatment	No	Ref	
	Yes	0.37 (0.36–0.38)	0.35 (0.34–0.36)
Frequency of CD4 testing^©^		Ref	
		0.21 (0.20–0.22)	0.19 (0.17–0.20)
Year of diagnosis	1989–2003	Ref	
	2004–2007	0.96 (0.94–0.98)	1.00 (0.95–1.04)
	2008-	1.02 (0.99–1.04)	1.33 (1.26–1.40)

^©^Time -varying covariate. ^©^Frequency of CD4 testing was defined as: the cumulative number of CD4 testing at each year divided by two (every six months).

**Table 4:** Fine and Gray model (hazard of the sub-distribution model) for Non-AIDS-related Death.

Variables		Non-AIDS-related Death	
		Crude HR (95%CI)	Adjusted HR (95%CI)
Nationality	Han	Ref	
	Uygur/Zhuang/Yi/Dai	1.26 (1.23–1.30)	0.86 (0.84–0.89)
	Others	1.13 (1.08–1.18)	0.88 (0.84–0.92)
Disease status^©^	HIV	Ref	
	AIDS	0.33 (0.32–0.34)	1.17 (1.14–1.20)
Possible Transmission route	Heterosexual	Ref	
	IDU	2.26 (2.19–2.32)	1.29 (1.25–1.33)
	Blood sell	0.24 (0.22–0.25)	0.32 (0.30–0.35)
	Homosexual	0.29 (0.27–0.32)	0.52 (0.48–0.56)
	Blood transfusion	0.58 (0.54–0.63)	0.64 (0.58–0.71)
	Sexual+IDU	1.22 (1.12–1.34)	1.04 (0.94–1.14)
	Others or unknown	1.95 (1.86–2.04)	1.20 (1.14–1.27)
Treatment	No	Ref	
	Yes	0.12 (0.11–0.12)	0.29 (0.27–0.30)
Frequency of CD4 testing^©^		Ref	
		0.14 (0.13–0.16)	0.20 (0.17–0.23)
Year of diagnosis	1989–2003	Ref	
	2004–2007	0.80 (0.78–0.82)	1.02 (0.96–1.09)
	2008-	1.37 (1.33–1.40)	1.59 (1.49–1.69)

^©^Time -varying covariate. ^©^Frequency of CD4 testing was defined as: the cumulative number of CD4 testing at each year divided by two (every six months).
