# Supplementary material for: Mortality among People Living with HIV and AIDS in China: Implications for Enhancing Linkage
Source: Sci Rep. 2016 Jun 21;6:28005. doi: 10.1038/srep28005 (PMC4914945; doi:10.1038/srep28005)
Supplement: Supplementary Information [file srep28005-s1.pdf]

Mortality among People Living with HIV and AIDS in China: Implications for Enhancing Linkage

Meng Li,[1,a] Weiming Tang,[2,a] Kai Bu, [1] Tanmay Mahapatra,[3] Xiayan Zhang,[1] Yibing Feng,[1] Fangfang Chen,[1] Wei Guo,[1] Liyan Wang,[1] Zhengwei Ding,[1] Qianqian Qin,[1] Shiliang Liu, [1] Joseph D. Tucker[2], Lu Wang, [1,*] ,Ning Wang [1]

[1]National Center for AIDS/STD Control and Prevention, Chinese Center for Disease Control and Prevention, Beijing, China;

[2]University of North Carolina Project-China, Guangzhou, China; [3]University of California, Los Angeles, CA, USA

Meng Li and Weiming Tang contributed equally to this work and are co-first authors

Correspondence:

Wang Lu

National Center for AIDS/STD Control and Prevention,

Chinese Center for Disease Control and Prevention,

Beijing, China

E-mail:wanglu64@163.com

Supplementary Table 1: Fine and Gray model (hazard of the subdistribution model) for AIDS-related Death among MSM Cases in China, 1989-2013 (N=32,958)

Variables		AIDS-related Death	
		**Crude HR(95%CI）**	**Adjusted HR（95%CI）**
Nationality	Han	Ref	
	Uygur/Zhuang/Yi/Dai	1.01(0.45-2.25)	0.52(0.25-1.11)
	Others	1.16(0.78-1.73)	1.12(0.75-1.69)
Occupation	Government staff	Ref	
	Farmer	2.70(2.15-3.40)	1.57(1.19-2.09)
	Housekeeping, housework and unemployed	1.27(1.04-1.55)	1.36(1.08-1.72)
	Worker	1.08(0.86-1.36)	1.14(0.87-1.50)
	Business	0.59(0.45-0.76)	0.83(0.62-1.11)
	Migrant workers	2.08(1.51-2.86)	1.50(1.05-2.15)
	Retired	3.60(2.51-5.15)	1.12(0.71-1.77)
Education	College or above	Ref	
	Illiteracy	5.18(2.58-10.39)	2.89(1.27-6.56)
	Elementary	3.35(2.65-4.25)	2.59(1.88-3.56)
	Junior high school	1.92(1.64-2.26)	2.03(1.59-2.59)
	Senior high school	0.82(0.69-0.98)	1.37(1.06-1.75)
Disease status©	HIV	Ref	
	AIDS	4.24(3.54-5.08)	16.77(13.41-20.96)
Treatment	No	Ref	
	Yes	0.53(0.45-0.62)	0.27(0.22-0.33)
Frequency of CD4 testing©		Ref	
		0.23(0.20-0.27)	0.17(0.14-0.20)
Year of diagnosis	1989-2003	Ref	
	2004-2007	1.07(0.82-1.40)	0.88(0.34-2.27)
	2008-	0.89(0.69-1.14)	0.82(0.32-2.07)

©Time -varying covariate.
